# A Comparative Study of LC-MS and FIA-(ESI)MS for Quantitation of *S*-Allyl-L-Cysteine in Aged Garlic Supplements

**DOI:** 10.3390/foods13172645

**Published:** 2024-08-23

**Authors:** Ignacio Jiménez-Amezcua, Marina Díez-Municio, Ana Isabel Ruiz-Matute, Ana Cristina Soria

**Affiliations:** 1 Instituto de Química Orgánica General (IQOG-CSIC), Juan de la Cierva 3, 28006 Madrid, Spain; ijimenez@iqog.csic.es (I.J.-A.); ana.ruiz@csic.es (A.I.R.-M.); 2 Pharmactive Biotech Products S.L.U., Faraday 7, 28049 Madrid, Spain; mdiez@pharmactive.eu

**Keywords:** food supplements, aged garlic, *S*-allyl-L-cysteine (SAC), LC-MS, FIA-(ESI)MS, frauds, quality, genuineness

## Abstract

The increasing consumption of food supplements demands the development of improved analytical methodologies to ensure their quality and authenticity. In this paper, two new approaches, liquid chromatography coupled to mass spectrometry (LC-MS) and flow injection analysis-(electrospray ionization) mass spectrometry (FIA-(ESI)MS), were optimized and validated for their application in the quantitative analysis of bioactive *S*-allyl-L-cysteine (SAC) in commercial aged garlic supplements (AGS). Although both methodologies were found to be useful for the sensitive and precise quantitation of SAC, the LC-MS approach allowed the differential determination of SAC and its bioactive diastereoisomer, *S*-1-propenyl-L-cysteine (S1PC), together with the identification of a number of organosulfur compounds typical of garlic. Mass fingerprints by FIA-(ESI)MS were proposed as an advantageous alternative to LC-MS analysis when the fast (4 min/sample) screening of AGS for their SAC content is intended, as in applications aimed at high-throughput quality control or standardization. Finally, the results gathered by the application of these two methodologies evidenced the highly variable composition of commercial AGS, as well as the identification of a number of potential composition frauds affecting their genuineness and benefits on health.

## 1. Introduction

Awareness of the proven benefits of a healthy lifestyle on quality of life and life expectancy has noticeably increased over the last few years. In this sense, the concern about the rising number of health problems associated with aging and lifestyle-related diseases (e.g., cardiovascular affection, overweight, obesity), particularly in so-called developed countries, has promoted the consumption of food supplements that, together with a balanced diet and regular physical exercise, may contribute to good health status.

Among a large number of natural sources, plant-based dietary supplements are in high demand by consumers as they are commonly perceived as harmless alternatives to synthetic drugs, providing bioactives with a wide spectrum of physiological effects [1,2]. Moreover, the easy accessibility of these products, not only through traditional stores but also through the online market, has contributed to expanding their consumption and increasing the economic impact associated with their production and commercialization. However, the limited availability of some sources, scarcely developed legal frameworks, and shortcomings of the current official protocols for ensuring their standardization and quality have given rise to a number of alerts and fraudulent practices involving food supplements [3]. Among others, discrepancies regarding the presence and content of the declared bioactives [4], the undeclared use of synthetic bioactive compounds [5], the replacement of the declared natural source with a cheaper alternative [2], and the addition of pharmacologically active compounds to enhance bioactivity or counteract undesired side effects have been reported for some supplements [6]. 

Garlic (*Allium sativum* L.) is widely used as a condiment in traditional cuisine [7,8], and its beneficial health properties have been considered in the development of a number of garlic-based supplements with different formulations (e.g., oils, extracts, and dehydrated garlic powder). However, the strong smell and taste of garlic, as well as its difficult digestion, may negatively affect consumer acceptance of these products. This has led to the development and marketing of supplements based on deodorized garlic and the use of alternative sources, such as black garlic, with better sensory characteristics. 

Black garlic is typically obtained by aging raw garlic bulbs at high temperatures (60–90 °C) under controlled humidity (70–90%) for 1–3 months [9,10,11,12]. This thermal processing induces non-enzymatic browning reactions (e.g., Maillard reaction), caramelization, and chemical oxidation, resulting in a product with a brown or even black color (depending on the processing conditions), sticky jelly-like texture, sweet flavor, and improved bioactive properties [8,13] compared to raw garlic. Other aging processes less commonly used in the food supplement industry involve the soaking of fresh garlic in hydroalcoholic solutions (e.g., 15–20% ethanol) at room temperature for extended periods of time (at least 18 months) [14] or fermentation of garlic with probiotics (*Saccharomyces cerevisiae*, *Monascus pilosus*, *Lactobacillus plantarum*, etc.) [15,16,17,18]. These alternative processes have been reported to preserve or even increase the concentration of garlic bioactives [15,17,18] and give rise to lower levels of unwanted compounds resulting from sugar degradation, such as hydroxymethylfurfural (HMF) [19].

Numerous studies have described the diverse and well-established bioactive properties of aged black garlic, including cardiovascular, antioxidant, anti-inflammatory, anticancer, immunomodulatory, hepatoprotective, neuroprotective, hypolipidemic, and hypoglycemic activities, among others [8,13,20,21,22]. These bioactivities have been reported to be mainly attributed to the organosulfur compounds (OSC) present in black garlic. Among them, *S*-allyl-L-cysteine (SAC), a water-soluble sulfur-containing amino acid arising from γ-glutamyl-*S*-allyl-L-cysteine (γ-GSAC) hydrolysis by γ-glutamyl transpeptidase enzyme [8,23], is the major OSC in aged black garlic. Its content, 4.5–10 times higher as compared to that of fresh garlic [12,24], is usually considered for standardization and valorization of commercial aged garlic supplements (AGS).

A number of analytical methodologies based on gas chromatography coupled with mass spectrometry (GC-MS) or liquid chromatography (LC) with different detectors have been reported for SAC analysis. Approaches based on GC-MS require prior derivatization of SAC, and tert-butyldimethylsilyl (TBDMS) derivatives have been shown to be advantageous for this purpose [14,25]. LC-UV methods allow the direct analysis of SAC, but the limited UV absorption of this bioactive compound, together with the common presence of spectral interferences associated with co-extracted compounds, hamper their use for the reliable quantitation of SAC in black garlic extracts [24,26]. In this sense, the advantages regarding the separation of SAC from other interferences and the sensitivity and selectivity of detection provided by reverse-phase LC-MS methods support their common use for SAC quantitation [14].

Flow injection analysis–mass spectrometry (FIA-MS) fingerprinting has recently been described as a high-throughput alternative to chromatographic methods for applications dealing with food classification and authentication [27,28,29,30,31]. However, FIA-MS approaches have not yet been applied to the analysis of AGS. Thus, in the present paper, the performance of two analytical methods by LC-MS and FIA-MS, after their corresponding optimization and validation, will be compared for the intended quantitation of SAC in commercial AGS. The advantages and limitations of both methodologies as fast and cost-effective approaches for the reliable quantitation of this bioactive, as well as for the assessment of AGS quality and genuineness, will also be evaluated here.

## 2. Materials and Methods

### 2.1. Reagents and Samples

Analytical standards (purity > 99%) of HMF, *S*-1-propenyl-L-cysteine (S1PC), and γ-GSAC were acquired from Sigma-Aldrich (St. Louis, MO, USA), and SAC from Tokyo Chemical Industry (Zwiindrecht, Belgium). Milli-Q water was obtained from an Advantage A10 system (Millipore, Bedford, MA, USA), while methanol (MeOH) and formic acid were acquired from Fisher Chemical (Newington, NH, USA).

Aged garlic supplements (AGS1-24) under study were purchased online from different websites and analyzed prior to their expiration date. Table S1 of the Supplementary Material shows their different formulations and compositions, as declared in the labeling.

Reference aged black garlic powdered extract Abg+^®^ (AGRef, ≥0.1% SAC) was provided by Pharmactive Biotech Products S.L.U. (Madrid, Spain), and was obtained using the Abg Cool-Tech^TM^ registered methodology.

### 2.2. LC-MS Analysis

#### 2.2.1. Sample Preparation

Ultrasound-assisted extraction of AGS and AGRef (100 mg) with 1 mL of Milli-Q water (Millipore, Bedford, MA, USA) was carried out (*n* = 3) using an ultrasonic bath (37 kHz) (Elma Schmidbauer GmbH, Singen, Germany) set at 40 °C for 25 min. Extracts thus obtained were further centrifuged at 4400× *g* for 5 min, and the supernatants were filtered through 0.2 µm teflon filters (Interchim, Montluçon, France) and diluted (1:10–1:50, *v*/*v*), as required. All extracts were frozen (−20 °C) and protected from light until analysis.

#### 2.2.2. Optimization of LC-MS Operating Conditions

Qualitative and quantitative analyses of SAC and other AGS components were performed on an Agilent 1100 series system, including an autosampler, a quaternary pump, a thermostated column compartment, and a diode array detector coupled to a 6125 single quadrupole mass spectrometry detector (Agilent Technologies, Santa Clara, CA, USA) provided with an electrospray ionization (ESI) source. According to Jiménez-Amezcua et al. [12], chromatographic separation was carried out using an InfinityLab Poroshell 120 Bonus-RP column (150 mm × 4.6 mm, 2.7 μm; Agilent Technologies) thermostatized at 30 °C, and flow rate was set at 0.4 mL min^−1^. A binary gradient consisting of water (eluent A) and MeOH (eluent B), both containing 0.1% formic acid, was used as the mobile phase. The injection volume was set at 5 μL.

Optimization of ESI parameters, considering both positive (ESI^+^) and negative (ESI^−^) polarities, was performed by infusion of a SAC standard solution (0.01 mg mL^−1^) under the conditions provided by a Box–Behnken experimental design (15 experiments in randomized order, Table S2 of Supplementary Material). Three independent variables were considered in the experimental design: the nebulizing gas pressure (N_2_, 99.5%) (*P*), nebulizing temperature (*T*), and fragmentor voltage (*FV*). The experimental ranges evaluated for each of these variables were *P* = 20–60 psi, *T* = 150–300 °C, and *FV* = 40–300 V.

Response surface methodology (RSM) (StatGraphics Centurion XVI software, Statistical Graphics Corporation, Rockville, MD, USA) was used to calculate the coefficients to maximize the response variable (SAC peak area) under positive and negative polarities (*R_SAC(+)_* and *R_SAC(−)_*, respectively) and their statistical significance, as well as to estimate the prediction errors (estimation standard error (SSE) and mean absolute error (MAE)). The quadratic model proposed was as follows:

*R_SAC_* = β_0_ + β_1_ *P* + β_2_ *T* + β_3_ *FV* + β_1,1_ *P*^2^ + β_2,2_ *T*^2^ + β_3,3_ *FV*^2^ + β_1,2_ *P·T* + β_1,3_ *P·FV* + β_2,3_ *T·FV* + ε, (1)

where β_0_ was the intercept, β_i_ were the first-order coefficients, β_i,i_ were the quadratic coefficients for the *i*^th^ factor, β_i,j_ were the coefficients for the interaction between factors i and j, and ε was the error. Under optimal *P*, *T*, and *FV*, capillary voltage (*CV*) was also optimized (3000–4000 kV) for a flow rate (*F*) set at 12 L min^−1^.

Mass spectra were acquired in SCAN mode (100–1000 *m*/*z*) and in SIM mode, registering the quasimolecular ion of SAC under positive (*m*/*z* 162 = [M + H]^+^), and negative (*m*/*z* 160 = [M − H]^−^) polarities. Data acquisition and processing were performed using HP Chemstation Rev. B.04.02 software (Agilent Technologies, Santa Clara, CA, USA).

Compound identification was based on chromatographic retention and MS data and was confirmed, when possible, by co-injection of the corresponding commercial standards. Quantitative analysis of SAC was carried out in triplicate using an external standard calibration curve (0.0001–0.05 mg mL^−1^).

### 2.3. Optimization of FIA-(ESI)MS Analysis

Mass spectral fingerprinting of SAC was carried out using the system previously described in Section 2.2.2., by replacing the chromatographic column with a polyetheretherketone (PEEK) tube (105 × 0.17 mm). FIA was used for the direct injection of the sample (5 μL for 4 min) into the carrier and further transmission into the ESI interface of the mass spectrometer. A hydroalcoholic mixture consisting of H_2_O:MeOH (93:7, *v*/*v*), both with 0.1% formic acid, was employed as the carrier. The flow rate was set at 0.4 mL min^−1^.

The ESI interface was operated in SIM mode under the polarity and operating conditions previously optimized in Section 2.2.2. All the analyses were performed in triplicate, and quantitation was performed following an external standard calibration curve of SAC (0.0001–0.05 mg mL^−1^).

### 2.4. Validation of LC-MS and FIA-(ESI)MS Methods

Different parameters were considered in the validation of the previously optimized methods for SAC quantitation. Precision was measured in terms of intra- and inter-day precision by analyzing two aged garlic supplements with different SAC concentrations (AGS2 and AGS22) within the same day (*n* = 5) or in 5 consecutive days, respectively. The linearity of response was assessed in the 0.0001–0.05 mg mL^−1^ range for both techniques by means of the coefficient of determination (R^2^). Limits of detection (*LOD*) and quantitation (*LOQ*) were calculated for SAC as three and ten times the signal-to-noise ratio (*S*/*N*), respectively. Recovery was calculated after spiking AGS2 and AGS22 extracts with SAC standard solutions at three concentration levels (in the range 0.005–0.01 mg mL^−1^ for AGS2 and 0.0025–0.0075 mg mL^−1^ for AGS22). The matrix effect was evaluated by quantifying SAC in AGS2 and AGS22 after dilution with water at different ratios (up to 1:100, *v*/*v*).

### 2.5. LC Coupled to Isotope Ratio Mass Spectrometry (LC-IRMS)

Prior to LC-IRMS analysis of the selected AGS, a clean-up step by solid-phase extraction (SPE) was required. AGS were re-dissolved in 10 mL of Milli-Q water (Millipore), sonicated for 10 min, and centrifuged at 1000× *g* for 45 min to separate the insoluble fraction. The supernatant was then passed through an SPE column prepared by filling a glass syringe (25 mL) with 5 g of a cation exchange resin (Dowex^®^ 50 WX 8, H^+^ form, 50–100 mesh, Merck KGaA, Darmstadt, Germany) following the procedure described by Kubec et al. [32]. The resin was conditioned with 10 mL of HCl (3%, *v*/*v*) and equilibrated with 10 mL of water. 1–2 mL of sample solution were loaded onto the cartridge, and extracts were purified with 10 mL of water. SAC was recovered with 40 mL of NH_4_OH (1M). Finally, the purified extracts were freeze-dried and re-dissolved in 1 mL of Milli-Q water (Millipore) for instrumental analysis.

LC-IRMS analysis of SAC in selected AGS, AGRef, and synthetic SAC standards was carried out on a Surveyor HPLC system (Thermo Fisher, Bremen, Germany) using a Primesep A LC column (100 Å, 2.1 × 250 mm, SIELC Co., Wheeling, IL, USA). Separation was carried out using a binary gradient of water (A) and 0.3% H_2_SO_4_ (B) as follows: 40% B to 100% B for 30 min. The flow rate and temperature were set at 0.3 mL min^−1^ and 30 °C, respectively. Aliquots of 2–5 μL were injected.

A Delta V Advantage isotope ratio mass spectrometer coupled to an LC-Isolink^TM^ interface (both from Thermo Fisher) was used for isotopic characterization (δ^13^C) of SAC. Organic compounds eluted from the LC system were oxidized online using the LC-Isolink™ interface. The effluent was combined with oxidation (50 g L^−1^ of sodium persulfate) and acid (10% phosphoric acid) reagents and heated at 100 °C. The CO_2_ produced in the oxidation reactor was extracted online using a TeflonAF™ membrane (DuPont, Wilmington, DE, USA) and directed into the IRMS system with a stream of He gas. The He flow was dried using Nafion™ (The Chemours Company, Wilmington, DE, USA) membranes before entering the IRMS instrument. Calibration of the IRMS system was performed by applying pulses of CO_2_ of a known isotopic composition along the LC run. The standard precision of the δ^13^C data provided by the LC-Isolink-IRMS instrument was 0.7‰.

### 2.6. Statistical Analysis

Statgraphics Centurion XVI software (Statistical Graphics Corporation) was used for the statistical analysis of data. The agreement between the declared values of SAC and the experimental concentrations determined either by LC-MS or FIA-(ESI)MS analysis was assessed using the *t*-test for independent samples (*p* < 0.05).

## 3. Results and Discussion

### 3.1. Development and Validation of LC-MS and FIA-(ESI)MS Methods for SAC Quantitation in AGS

#### 3.1.1. Optimization of (ESI)MS Operating Conditions

Prior to comparing the performance of LC-MS and FIA-(ESI)MS methods for the intended quantitation of bioactive SAC in AGS, the ESI parameters, which are common to both techniques, were optimized.

After preliminary FIA-(ESI)MS experiments in SCAN mode, *m*/*z* 162 ([M + H]^+^) and *m*/*z* 160 ([M − H]^−^) were shown to be the most abundant adducts under positive and negative polarities, respectively, and were therefore considered for further data acquisition under SIM mode.

**Figure 1:**
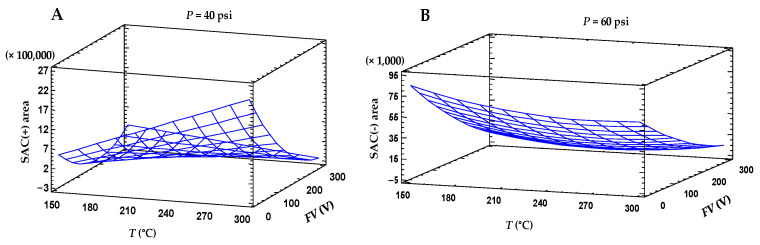
Surface plots of the Box–Behnken experimental design aimed to optimize the ESI parameters under (**A**) positive and (**B**) negative polarities for maximization of *S*-allyl-L-cysteine (SAC) area.

The set of 15 experiments provided by the Box–Behnken experimental design described in Section 2.2.2, including different *T*, *FV,* and *P* combinations, was considered for the maximization of the SAC response [*R_SAC(+)_* and *R_SAC(−)_*]. The experimental results obtained under positive and negative polarity (peak areas for *m*/*z* 162 and for *m*/*z* 160, respectively) are shown in Table S2. The coefficients of the quadratic models proposed for maximization of *R_SAC(+)_* and *R_SAC(−)_* and their statistical significance were calculated using a response surface methodology (Figure 1). As for the *R_SAC(+)_* model, *FV*, *T·FV*, and *FV*^2^ were the factors statistically significant at the 95% confidence level. The model equation, the goodness of the fitting and the prediction errors were, respectively, *R_SAC(+)_* = −5.2 × 10^5^ + 8.5 × 10^3^*T* − 0.8 × 10^3^*FV* + 3.5 × 10^−10^*T*^2^ − 50.02*T·FV* + 24.8*FV*^2^ + 2.04 × 10^−10^*FV·P* + 1.16 × 10^−10^*P*^2^, R^2^ = 89%, SSE = 309,417 and MAE = 141,152. The maximum response estimated under optimal ESI+ conditions (*T* = 300 °C, *FV* = 40 V, *P* = 60 psi) was *R_SAC(+)_* = 1.84 × 10^6^.

As for the model obtained under negative polarity (*R_SAC(−)_* = 1.2 × 10^5^ − 0.9 × 10^3^*T* − 0.3 × 10^3^*FV* + 2 × 10^3^*P* + 1.64*T*^2^ + 0.50*T·FV* + 0.57*T·P* + 0.31*FV*^2^ − 2.50*FV·P* − 17.83*P*^2^, R^2^ = 91%; SSE = 9909; MAE = 4734), *FV* was the only significant (*p* < 0.05) factor. This model provided the maximum response (*R_SAC(−)_* = 70,745) under optimal ESI conditions (*T* = 150 °C, *FV* = 40 V, *P* = 55 psi).

From the comparison of both datasets, it was found that although the optimal ESI operating conditions were dependent on the polarity considered, the common setting of *FV* = 40 V was required to avoid the easy fragmentation of SAC experimentally observed and give rise to a decrease in response. Under these optimal *T*, *FV,* and *P* conditions, the effect of the capillary voltage (*CV*) within the range 3000–4000 V was also considered for the optimization of *R_SAC(+)_* and *R_SAC(−)_*. Although *CV* was not relevant for *R_SAC(−)_*, the sensitivity of *R_SAC(+)_* increased significantly (up to eight times) when *CV* was set at 3000 V. Finally, when *R_SAC(+)_* and *R_SAC(−)_* were compared, the response under positive polarity was found to be 26 times higher than that in ESI mode. Therefore, the setting of *CV* at 3000 V, together with the previously mentioned optimal ESI^+^ conditions, were selected for further validation of a sensitive method for SAC quantitation. Under these conditions, the calculated and experimental responses were very similar (RSD = 4%).

#### 3.1.2. Validation

The analytical parameters considered in the validation of the LC-MS and FIA-(ESI)MS methods previously developed are shown in Table 1. As can be seen, both methods exhibited good linearity (R^2^ > 0.995) within the SAC concentration range expected for the AGS under study, with LC-MS showing a wider linearity range (0.0001–0.05 mg mL^−1^). In terms of sensitivity, the method by FIA-(ESI)MS outperformed the LC-MS method, as evidenced by a lower *LOD* (0.84 vs. 1.19 ng mL^−1^, respectively) and *LOQ* (2.78 vs. 3.98 ng mL^−1^, respectively). As compared to the literature, the sensitivity of the LC-MS (SIM) and FIA-(ESI)MS (SIM) methods developed here was significantly higher than that of LC methods with UV or fluorescence detection previously reported (*LOD*: 29.67 and 6.28 μg mL^−1^, respectively) [24]. However, it was in the order of methodology based on liquid chromatography-tandem mass spectrometry (LC-MS/MS) under the multiple reaction monitoring (MRM) mode described by Woo et al. [17] (*LOD*: 60 ng mL^−1^).

Regarding the intra- and inter-day precision of these methods, evaluated over two AGS standardized at two different SAC concentrations (0.1 and 1% for AGS2 and AGS22, respectively), the results obtained were similar for both techniques (RSD < 10%). These results are also in good agreement with precision data previously reported for other LC methodologies based on the use of different detection systems [17,24]. As regards accuracy, recoveries obtained by both techniques by spiking AGS2 and AGS22 with known amounts of SAC were also good (LC-MS: 102–112% and FIA-(ESI)MS: 105–106%). Finally, the potential matrix effect in the analysis of AGS was ruled out for both techniques, as no significant differences at the 95% confidence level were found in the SAC content determined in the original extract and after its dilution in the range 1:20–1:100 for FIA-(ESI)MS and 1:2–1:20 for LC-MS.

### 3.2. SAC Content of Commercial AGS

The previously optimized and validated LC-MS and FIA-(ESI)MS methods were further applied to the quantitation of SAC in 24 commercial AGS and in AGRef. The experimental results obtained by both techniques, together with the SAC content declared in the labeling of these products, are shown in Table 2.

A good match between the results obtained by LC-MS and FIA-(ESI)MS was found for most of the samples under study (e.g., AGS18, AGS21, and AGS24), with 29% of the analyzed supplements (AGS3, AGS5, and AGS10-14) equivalent to 88% of non-SAC-standardized AGS, showing levels of SAC below the *LOQ* of both methodologies. Although the standardization in SAC was not detailed for most of these supplements, the experimental results obtained here would question the potential health benefits associated with their consumption. Moreover, composition fraud, if other reasons are ruled out, would be responsible for the discrepancies observed regarding the SAC content of AGS3 and AGS12.

As for the SAC-standardized supplements, 50% showed good agreement between the SAC content experimentally determined and that declared in the labeling (e.g., AGS1, AGS6, AGS7, AGS16, etc.), whereas significant (*p* < 0.05) differences were found for the remaining AGS under study. Within this last group, SAC-standardized AGS showing a bioactive content lower than expected (e.g., AGS3, AGS8, AGS12, and AGS21) would represent a potential labeling fraud.

### 3.3. Characterization of AGS Composition

**Figure 2:**
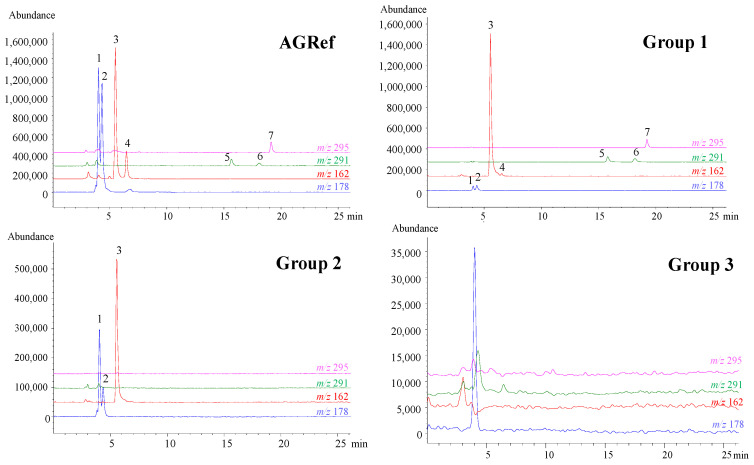
LC-MS profiles of AGRef, AGS4 (Group 1), AGS1 (Group 2) and AGS10 (Group 3). Traces of *m*/*z* 178 (blue), 162 (red), 291 (green), and 295 (pink) correspond to [M + H]^+^ adducts of (1) cycloalliin, (2) alliin, (3) SAC, (4) S1PC, (5) γ-GSAC, (6) γ-GS1PC, and (7) Glu-Phe.

In order to gain insight into the potential composition or labeling frauds identified in Section 3.2. regarding the SAC content of the commercial AGS under study, the LC-MS profiles of these samples were compared with that of the AGRef extract. As can be seen in Figure 2, supplements were grouped into three different classes according to their similar composition. Supplements of Group 1 (AGS4, AGS6, AGS8, AGS90, AGS19, and AGS20) were considered to be genuine as they were characterized by the presence of a number of organosulfur compounds, including SAC, its diastereoisomer S1PC, as well as their precursors, γ-GSAC, and γ-glutamyl-*S*-1-propenyl-L-cysteine (γ-GS1PC) [23,33]. Other OSC present in these samples were alliin and its isomer, cycloalliin [12,34], and the dipeptide glutamyl-phenylalanine (Glu-Phe), all of which are typical of black garlic [12,33], and were experimentally detected in the reference sample used here (AGRef).

Compared to Group 1, supplements included in Group 2 showed a simpler LC-MS profile, in which either SAC (e.g., AGS1, AGS7, AGS18, and AGS22-24) or both SAC and S1PC in a wide range of concentrations (AGS2, AGS15-17, AGS21) were the most abundant OSC detected. Although the required presence of SAC would support the intended physiological effect associated with their consumption, the genuineness of these supplements would be questionable due to the lack of other typical aged garlic compounds. Therefore, Group 2 samples were presumably considered supplements in which SAC of non-natural origin was used for manufacturing and evaluation of SAC isotopic composition by LC-IRMS analysis (Section 3.4) was found to be required for further confirmation of this hypothesis.

Lastly, supplements with no SAC content were grouped together in Group 3 (AGS3, AGS5, and AGS10-14). These supplements predominantly contained low molecular weight carbohydrates such as mono-, di-, and trisaccharides, probably arising from the antiaggregants used for their formulation, and compounds derived from sugar degradation such as HMF. In this case, fraud is committed not only because the desired physiological effect associated with bioactive SAC is prevented but also because supplements marketed as standardized in SAC and not complying with the labeling represent composition fraud (e.g., AGS3 and AGS12).

A noticeable advantage of the developed LC-MS method is the ability to differentiate between SAC and its diastereoisomer S1PC, to which a number of bioactive properties (e.g., antioxidant, cardioprotective, etc.) have been ascribed [21,23]. This differentiation was not possible because the FIA-(ESI)MS configuration used here as a single quadrupole mass spectrometer does not discriminate compounds with identical nominal mass. As shown in Table 3, 42% of the AGS under study contained variable amounts of S1PC in addition to SAC. These contents, normalized to the corresponding SAC concentration, were below 3% in most cases and close to that of AGRef (11%). However, for samples AGS4, AGS9, and AGS17, these relative values were noticeably higher (in the range of 72–93%).

A detailed evaluation of the available information related to the processing of some of these samples (e.g., AGS4 and AGS9) revealed that they had not been aged by a conventional heating process but by soaking in hydroalcoholic solutions at room temperature [17]. This procedure has been described to give rise to a white-colored product, different from the typical brown-black aged garlic usually appreciated by consumers, with high contents of both SAC and S1PC. Alternatively, it has also been reported that anaerobic fermentation of garlic using different microorganisms (e.g., *S. cerevisiae*, *Bacillus subtilis*, etc.) leads to highly variable proportions of γ-GSAC and γ-GS1PC, which are precursors of SAC and S1PC, respectively [15,16,17,18]. Although a greater number of AGS from different garlic varieties, locations, etc., and aged under known conditions should be analyzed to confirm these results, the information provided by LC-MS seems to be useful not only to determine the differential content of these two bioactive compounds in AGS but also to identify those that have presumably undergone a non-conventional aging process.

As regards AGS17, for which information on processing is not available, confirmation of its potential non-genuine origin supported by the results on OSC previously mentioned would require that a non-selective synthetic procedure providing the similar yields of SAC and S1PC experimentally determined is considered (see Section 3.4).

On the other hand, the results shown in Table 3 were also used to evaluate the extent that which S1PC may interfere with the quantitation of SAC by FIA-(ESI)MS, as compared to LC-MS analysis. Thus, considering the similar MS response of both bioactives, an overestimation of SAC by FIA-(ESI)MS would be expected for AGS showing either a high S1PC content and/or a high relative abundance of this bioactive compared to SAC. However, although this effect was evidenced for some of the samples under study (e.g., AGS2, AGS4, and AGS9), it was not clear for others (particularly for those with the lowest content of these two bioactives). Therefore, the usefulness of FIA-(ESI)MS as a rapid (4 min/sample) tool for screening SAC in AGS is demonstrated, and a particularly good match with LC-MS data will be obtained when this approach is applied to AGS processed under conventional aging procedures, usually giving rise to low S1PC contents.

### 3.4. Non-Natural SAC in Commercial AGS

LC-IRMS has been proven to be useful for a number of applications in food authentication [35,36,37] and, as regards garlic, has previously been described for the successful classification of samples from different Italian regions [37] or Asian countries [38]. However, no previous reference has addressed its use for the assessment of the natural origin of SAC present in AGS, and therefore, it was considered here for confirmation of the genuineness results previously described (Section 3.3).

Four samples from both Group 1 and Group 2 were selected for these analyses. AGS4 and AGS8 were considered representative supplements classified as genuine based on their OSC-rich chromatographic profiles and similitude to AGRef. In contrast, AGS17 and AGS23 were chosen as presumably non-genuine supplements enriched with non-natural (either synthetic or biotechnologically produced) SAC. The isotopic compositions of these samples determined by LC-IRMS, together with that of a synthetic SAC standard and AGRef (natural reference), are shown in Table 4.

The variability observed for the isotopic composition of SAC in the samples under study (δ^13^C data ranging from −28.0 to −20.4‰) allowed their effective classification. Thus, δ^13^C data for AGS8 and AGS4 (−27.9 and −27.3‰, respectively) were close to the δ^13^C value for the natural SAC reference (−28.0‰) and were in line with data previously reported by different authors for *Allium sativum*, the C_3_ pathway plant expected to be used for manufacturing AGS (−29.9 to −24.4‰, [38]; −28.5 to −25.6‰, [37]); average δ^13^C = −27‰, [39]). Therefore, in agreement with the conclusions drawn from the LC-MS analysis, the genuineness of the AGS included in Group 1 was assured.

The δ^13^C data for AGS17 and AGS23 (−20.4‰) were far from those of genuine samples and were similar to the δ^13^C value determined for the synthetic SAC standard (−22.3‰). These results were also in agreement with previous studies in which synthetic compounds have been reported to exhibit δ^13^C values out of the range for natural compounds in vegetable matrixes [40,41,42]. Therefore, LC-IRMS data would support the use of non-natural SAC for the formulation of supplements included in Group 2, in agreement with the poor profiles obtained by LC-MS analysis. The dissimilarity observed with respect to the synthetic SAC could be attributed, among others, to the different carbon sources and procedures followed for the synthesis of this bioactive.

## 4. Conclusions

Two analytical methods by LC-MS and by FIA-(ESI)MS have been optimized and validated for the first time for the quantitative determination of *S*-allyl-L-cysteine in aged garlic supplements. Although both methodologies have been proven to allow the reliable quantitation of this bioactive, the LC-MS approach enables the discrimination between SAC and its diastereoisomer, S1PC, a bioactive detected in some of the AGS under study, and an additional quality marker whose content depends among others, on the garlic aging process.

Mass fingerprints by FIA-(ESI)MS are proposed as an advantageous alternative to the LC-MS approach when fast (4 min/sample) screening of AGS for their SAC content is intended and when no differentiation of SAC and S1PC is required. This would be the case for applications highly demanded by both industries of the nutraceutical sector or by regulatory bodies, such as the high-throughput quality control of AGS. In this sense, only those AGS samples that do not meet specifications or do not contain SAC would need to undergo in-depth studies using more time-consuming and expensive LC-MS approaches, thus contributing to the rational use of facilities for AGS authentication and quality control.

Finally, the application of both methodologies to 24 commercial AGS has revealed not only the wide variability regarding their SAC content but has also allowed the identification of a variety of potential composition frauds affecting compliance with labeling, presumed functionality, or genuineness. The results of this study contribute to the demand for improved methodologies and scientifically based data to ensure the quality and authenticity of AGS.

## Figures and Tables

**Table 1:** Validation of LC-MS and FIA-(ESI)MS methods for the analysis of SAC.

LC-MS Validation	AGS2 (1% SAC)	AGS22 (0.1% SAC)
Intra-day precision (RSD, %)	0.99	3.97
Inter-day precision (RSD, %)	3.54	7.36
Calibration curve	y = 5.43 × 10^7^x (R^2^ = 0.9948)	
Linear range (mg mL^−1^)	0.0001–0.05	
*LOD*/*LOQ* (ng mL^−1^)	1.19 (0.042)/3.98 (0.139) *	
Recovery (%)	112 (10.6)/102 (6.8) *	
**FIA-(ESI)MS Validation**	**AGS2** **(1% SAC)**	**AGS22** **(0.1% SAC)**
Intra-day precision (RSD, %)	3.57	1.96
Inter-day precision (RSD, %)	5.20	9.25
Calibration curve	y = 9.41 × 10^7^x (R^2^ = 0.9978)	
Linear range (mg mL^−1^)	0.0001–0.025	
*LOD*/*LOQ* (ng mL^−1^)	0.84 (0.01)/2.78 (0.03) *	
Recovery (%)	105 (4.1)/106 (9.8) *	

* Mean and standard deviation in brackets for *n* = 3.

**Table 2:** Content (%) of SAC in aged garlic supplements under study (AGS1-AGS24) and in the reference extract (AGRef) experimentally determined by LC-MS and FIA-(ESI)MS and SAC values declared in their corresponding labels.

Code	Experimental SAC (%) *		Declared SAC (%)
	LC-MS	FIA-(ESI)MS	
AGS1	0.106 (0.003) ^a^	0.157 (0.008) ^b^	0.1 ^a^
AGS2	0.89 (0.06) ^a^	0.96 (0.03) ^b^	1 ^b^
AGS3	tr **	tr	0.25
AGS4	0.108 (0.005)	0.162 (0.002)	- ***
AGS5	tr	tr	-
AGS6	0.148 (0.005) ^a^	0.082 (0.005) ^b^	0.1 ^b^
AGS7	0.143 (0.006) ^a^	0.092 (0.002) ^b^	0.1 ^b^
AGS8	0.829 (0.001) ^a^	0.68 (0.02) ^b^	1 ^c^
AGS9	0.083 (0.002)	0.1187 (0.0001)	-
AGS10	tr	tr	-
AGS11	tr	tr	-
AGS12	tr	tr	0.1
AGS13	n.d. **	n.d.	-
AGS14	tr	tr	-
AGS15	0.07 (0.01) ^a^	0.09 (0.01) ^b^	0.1 ^b^
AGS16	0.20 (0.01) ^a^	0.125 (0.001) ^b^	0.1 ^c^
AGS17	0.0169 (0.0002)	0.0084 (0.0001)	-
AGS18	0.073 (0.001) ^a^	0.074 (0.003) ^a^	>0.1 ^b^
AGS19	0.069 (0.003) ^a^	0.043 (0.002) ^b^	0.013 ^c^
AGS20	0.06 (0.01) ^a^	0.032 (0.002) ^b^	0.013 ^c^
AGS21	0.57 (0.03) ^a^	0.62 (0.01) ^a^	1 ^b^
AGS22	0.090 (0.003) ^a^	0.062 (0.001) ^b^	0.1 ^a^
AGS23	1.13 (0.04) ^a^	0.75 (0.11) ^b^	1 ^a,b^
AGS24	1.33 (0.02) ^a^	1.01 (0.35) ^a^	1 ^a^
AGRef	0.141 (0.005) ^a^	0.1105 (0.0003) ^b^	≥0.1 ^b^

^a–c^ Significant (*p* < 0.05) differences between experimental (by LC-MS and by FIA-(ESI)MS) and declared SAC content. * Average and standard deviation are in brackets (*n* = 3). ** tr, traces (<*LOQ*) and n.d., non-detected (<*LOD*). *** -, non-declared.

**Table 3:** Concentration of S1PC (in mg g^−1^ and as a percentage of SAC content) determined by LC-MS in the AGS under study.

Code	S1PC (mg g^−1^)	S1PC (% Relative to SAC)
AGS2	0.23 (0.01) *	3
AGS4	0.78 (0.12)	72
AGS6	0.111 (0.004)	7
AGS8	0.228 (0.001)	3
AGS9	0.7 (0.2)	91
AGS15	0.006 (0.001)	1
AGS16	0.011 (0.002)	1
AGS17	0.16 (0.01)	93
AGS19	0.004 (0.001)	1
AGS21	0.17 (0.02)	3
AGRef	0.16 (0.002)	11

* Average and standard deviation are in brackets (*n* = 3).

**Table 4:** Isotopic ratios of δ^13^C in selected AGS.

Sample	δ^13^C of SAC (‰)
Synthetic SAC standard	−22.3
AGRef (natural reference)	−28.0
AGS4	−27.3
AGS8	−27.9
AGS17	−20.4
AGS23	−20.4

## Data Availability

The original contributions presented in the study are included in the article/Supplementary Materials, further inquiries can be directed to the corresponding author.

## Supplementary Materials

The following supporting information can be downloaded at https://www.mdpi.com/article/10.3390/foods13172645/s1, Table S1: Composition of AGS under study; Table S2: Box–Behnken experimental design for optimization of ESI parameters in the LC-MS and FIA-(ESI)MS analysis of SAC in AGS.
