# Supplementary material for: A Comparative Study of LC-MS and FIA-(ESI)MS for Quantitation of S-Allyl-L-Cysteine in Aged Garlic Supplements
Source: Foods. 2024 Aug 23;13(17):2645. doi: 10.3390/foods13172645 (PMC11394311; doi:10.3390/foods13172645)
Supplement: Supplementary file 1 [file foods-13-02645-s001.zip › foods-3156921-supplementary.pdf]

Table S1. Composition of AGS under study.

Code	Formulation	Composition
AGS1	125 mg extract / capsule	Ingredients of ecological agriculture. ABG10+® extract (*Allium sativum* L.), **0.1% SAC**, maltodextrin, acacia fibers.
AGS2	120 mg extract /capsule	Aged black garlic extract (ratio 100:1), **1% SAC**. Bulking agent (corn starch and cellulose), anti-caking agent (magnesium stearate, magnesium silicate and silicon dioxide).
AGS3	500 mg extract / capsule	Black garlic extract (82%) including 4% of flavonoids, **0.25% SAC**, gelatin, anti-caking agents (silicon dioxide and rice extract), arabic gum and sunflower oil.
AGS4	300 mg extract / capsule	Aged garlic extract powder (bulb), whey, gelatine and magnesium stearate.
AGS5	600 mg extract / capsule	Black garlic extract 15:1 (bulb), rice starch.
AGS6	500 mg extract / capsule	Aged black garlic extract with dextrins (10:1) with 3% polyphenols, **0.1% SAC**, anti-caking agent (silicon dioxide).
AGS7	250 mg extract / capsule	ABG10+® extract (*Allium sativum* L. - maltodextrin), **0.1% SAC**, 150 mg citric bioflavonoids (with maltodextrins), hesperidin (60%), 12 mg of vitamin E (100% VRN), 200 µg of folic acid (vit. B9 100%). Anti-caking agents: calcium phosphate, α-tocopherol acetate, etc.
AGS8	120 mg extract / capsule	Dry extract of aged black garlic (*Allium sativum* L., bulb), **1% SAC**. Bulking agents: corn starch and cellulose. Anti-caking agents: magnesium stearate, magnesium silicate and silicon dioxide.
AGS9	600 mg extract / capsule	Aged garlic extract powder (bulb), gelatin, microcrystalline cellulose, magnesium stearate and silica.
AGS10	250 mg extract / capsule	Aqueous extract of fermented black garlic bulbs, gelatine 75 mg. Anti-caking agents: magnesium stearate 30 mg, talc 20 mg.
AGS11	250 mg black garlic / capsule	Powder of biological black garlic bulbs (50%), maltodextrin 100 mg. Anti-caking agents: talc 20 mg.
AGS12	450 mg black garlic / capsule	Fermented black garlic (*Allium sativum*), hydroxypropylmethylcellulose. 0.45 mg **SAC (0.1%)**, 10.75 mg polyphenols / capsule.
AGS13	187.5 mg extract /capsule	Black garlic extract 4:1 (*Allium sativum*), microcrystalline cellulose, silica and magnesium stearate.
AGS14	650 mg black garlic / capsule	Aged black garlic (*Allium sativum*) by enzymatic fermentation. Other ingredients: gelatin, rice bran, calcium palmitate, silica.
AGS15	250 mg extract / capsule	Black garlic extract (*Allium sativum*) ABG10+®, **0.1% SAC**, maltodextrin, microcrystalline cellulose, silicon dioxide, magnesium stearate.

AGS16	250 mg extract / capsule	Dry extract of black garlic (ratio 10:1, **0.1% SAC**, 3% polyphenols). Bulking agent: microcrystalline cellulose, magnesium stearate. Anti-caking agent: silicon dioxide.
AGS17	200 mg extract / capsule	Black garlic extract (*Allium sativum)* (ratio 10:1), rice powder.
AGS18	500 mg extract / tablet	Black garlic extract (50.2%) ABG10+®, **>0.1% SAC**. Bulking agents: arabic gum, rice flour. Anti-caking agent: lecithin, starch. Gelling agent: guar gum. Emulgent: glycerol.
AGS19	245 mg black garlic extract / capsule	Powder and extract of black garlic (*Allium sativum*) bulb, **SAC** (130 µg). Others: BIO apple (*Malus domestica*) fibers.
AGS20	245 mg black garlic extract / capsule	Powder (totum integral) and extract of black garlic (*Allium sativum*) bulb, **SAC** (130 µg). Others: BIO apple (*Malus domestica*) fibers.
AGS21	120 mg extract / capsule	Dry aged black garlic extract (*Allium sativum* L.) (ratio 100:1) and corn starch, **1% SAC**. Anti-caking agents: magnesium salts of fatty acids, magnesium silicate and silicon dioxide.
AGS22	200 mg extract / capsule	Dry black garlic extract (*Allium sativum*) (ratio 10:1, **0.1% SAC**) and maltodextrin.
AGS23	200 mg FBG22™ / capsule	Fermented black garlic bulb (FGB22™, *Allium sativum*) (200 mg extract: 2 mg SAC; **1% SAC**), ginger rhizome/root (fresh freeze dried) (100 mg *Zingiber officinale*) and vegetable capsule shell (hydroxypropyl methylcellulose).
AGS24	200 mg FBG22™ / capsule	Fermented black garlic bulb (FGB22™, *Allium sativum*) (200 mg extract: 2 mg SAC; **1% SAC**), ginger rhizome/root (fresh freeze dried) (100 mg *Zingiber officinale*) and vegetable capsule shell (hydroxypropyl methylcellulose).

Table S2. Box-Behnken experimental design for optimization of ESI parameters in the LC-MS and FIA-(ESI)MS analysis of SAC in AGS.

***T* (ºC)**	***FV* (V)**	***P* (psi)**	**SAC Area**	
			**ESI$^+$**	**ESI$^-$**
150	170	20	8825	26218
225	170	40	93448	21026
300	300	40	864	5728
150	40	40	52548	74059
225	170	40	81232	21094
300	170	20	48283	13411
150	300	40	267	6501
300	170	60	53645	20790
225	40	60	1108185	46440
225	300	20	745	3842
150	170	60	19631	30164
225	40	20	639815	18682
225	170	40	87881	19515
300	40	40	2003949	53716
225	300	60	889	5594
